# Pharmacies and primary care: a global development framework

**DOI:** 10.2471/BLT.19.248435

**Published:** 2020-06-02

**Authors:** Arit Udoh, Desak Ketut Ernawati, Mary Akpan, Kirsten Galbraith, Ian Bates

**Affiliations:** a School of Medical and Dental Sciences, University of Birmingham, Birmingham, England.; b Faculty of Medicine, Universitas Udayana, Denpasar, Indonesia.; c Faculty of Pharmacy, University of Uyo, Akwa Ibom State, Nigeria.; d Faculty of Pharmacy and Pharmaceutical Sciences, Monash University, 381 Royal Parade, Parkville 3052, Melbourne, Australia.; e University College London School of Pharmacy, UCL-FIP Collaborating Centre, London, England.

Pharmacists are a critical health-care workforce to attain the goal of universal health coverage (UHC) and the health-related sustainable development goal (SDG) 3,^1^ particularly in relation to optimizing safe, responsible and effective use of medicines. Pharmacists provide preventative and public health services and are a main access point to primary health care, particularly for people with acute and long-term conditions. The 2018 Astana Declaration emphasizes the importance of primary health-care services in achieving health for all and further underscores the key role pharmacists play in access and delivery of primary health care.^2^

A key objective of the World Health Organization (WHO) *Global strategy on human resources for health: workforce 2030*,^3^ is optimizing the performance, quality and impact of the health workforce through evidence-informed policies on human resources for health. This objective aligns with the call for investment in health workforce development so that “all health-care workers have skills that match the health needs of the population and can work to their full potential.”^3^ Attaining these high-level objectives will require reconfiguring the health workforce, including within the pharmaceutical sector, to ensure that health workers practice using all their skills, within the full scope of their practice.^4^ Population ageing, dynamic shifts in disease profiles, increasing burden of noncommunicable and long-term conditions, technological advancement and uptake in personalized medicine and advanced therapies are additional justifications for this objective.

The pharmaceutical workforce needs highly competent, specialized pharmacists providing expertise in areas of focus. However, in the global primary care context the need for a professionally recognized expert cadre of generalist pharmacists, with more advanced capabilities to meet patients’ needs and support UHC, is even more pressing. Developing a career pathway for pharmacists that is structured with clear signposts for development and is adaptable to all practice settings, particularly primary care settings, is therefore essential. Such pathway would ensure the availability of a workforce capable of meeting the increasingly complex medicines and health needs of their local communities.

In low- and middle-income countries, barriers to effective primary care pharmacy practice include weak health systems, unclear quality of pharmaceutical services provided in community pharmacies and limited post-registration (that is, after pharmacists register to practice) education and training.^5^ For example, in south-east Asia, where pharmacists play a key role in the provision of drug information and pharmaceutical services in community health centres, experience in Indonesia shows that pharmacists require additional training to improve their competence in delivering these services.^6^ In many sub-Saharan African countries, formal post-registration training and skill enhancement tools for developing advanced capabilities in pharmacy are lacking.^7^ Evidence and information on the pharmaceutical workforce in the WHO Eastern Mediterranean Region is sparse compared with other WHO regions. However, the existing literature does report persistent workforce challenges,^8^ the most significant being a lack of workforce planning and intelligence across the WHO Eastern Mediterranean Region.

This evidence, coupled with increasing societal challenges regarding access to primary health care and medicines expertise, suggests that advancing pharmacists’ role in primary health care in low-income countries requires policy support and enhanced leadership, in addition to advancement in workforce competency. A global need for planned and structured post-registration training to increase capabilities of pharmacists also exists. To achieve the primary health-care vision set out in the Astana Declaration^2^ and to tackle the increasing complexity of medicines management for long-term conditions, communicable disease and preventative community health, we need to ensure we have mechanisms available for recognized advancement of practice in medicines expertise as a significant component of UHC.

## Transformation for primary care

The International Pharmaceutical Federation supports pharmacists globally to improve access to primary health care. Through the Workforce Transformation Programme, the federation has set out a roadmap to facilitate the systematic transformation of the global pharmaceutical workforce by providing the appropriate strategic and evidence-based tools to support advancement and professional development.^9^ One component of this global roadmap is a set of 13 pharmaceutical workforce development goals, a globally consented framework to support and drive country-level workforce transformation based on population health-care needs. The fourth and fifth goals are advanced and specialist expert development and competency development respectively, and provide indicators for progress that is informed by published evidence and workforce development tools. The federation’s commitment to facilitating global implementation of these goals now includes provision of a framework for advanced practice development: the Global Advanced Development Framework.^4^ This framework complements the federation’s Global Competency Framework for foundation and early career practice, which is already being implemented at country level by professional leadership organizations.^10^ Previous investigation of utility of the global framework has demonstrated its applicability to practice in low- and middle-income countries, including Ghana, Kenya, Nigeria and South Africa,^7^ demonstrating the feasibility and relevance of a global set of competencies to country-level pharmacy practice.

Both frameworks represent validated infrastructure tools intended to support the professional development and advancement of the pharmaceutical workforce everywhere, to benefit patient care.^4^ In particular, the Global Advanced Development Framework is a matrix that maps three stages of broad-based advanced practice across a set of generic developmental competencies that can be adopted and adapted by countries, regions and professional organizations. These advanced competencies are organized in clusters relating to; (i) medicines expertise; (ii) leadership capabilities (for example, clinical, medicines related activities, teamwork, and others); (iii) managing health and professional delivery services and people; (iv) training and mentoring; and (v) developing evaluation skills and innovation in health and professional service provision.^4^ These capabilities are all common components of a rounded, flexible, effective and advanced pharmacist practitioner, and have applicability across all sectors, including in primary care. The Global Advanced Development Framework has been developed to support countries progress (via their national organizations and stakeholders) and to advance medicines-related practice. The framework is also designed for use by individual practitioners to map and plan their professional advancement and expand their personal development portfolio and career pathway.

## Regional and country experiences

Some countries, such as Australia and the United Kingdom of Great Britain and Northern Ireland, have established developmental frameworks to describe and recognize the advancement of their pharmaceutical workforce.^4^ However, these country cases represent high-income, anglophone nations. With the availability of the Global Advanced Development Framework, low- and middle-income countries can now develop locally driven advanced practice frameworks, in a fast-track adopt and adapt process, for example in Indonesia and Jordan.

In Indonesia, the Indonesian Pharmacists Association has identified a need to develop access to a professional recognition and advance practice pathway, particularly in primary care pharmacy settings, to support better access to medicines expertise. Following a comprehensive pharmacy workforce analysis, a needs-based approach has resulted in the Framework Apoteker Advance,^11^ with a four-stage workforce development model (early advanced, advanced stage 1, 2 and 3). Engagement with stakeholders with this framework has already led to agreement that the pharmacists’ association should recommend provision of solid framework guidelines for each sector of practice. This needs-based approach has also identified that Indonesian pharmacists practising at early advanced stage require additional foundation training, while pharmacists at advanced stages 1, 2 and 3, particularly community pharmacists, require a more structured advanced career pathway and continuing professional development.

Likewise, in the WHO Eastern Mediterranean Region, gaining better national-level commitments to pharmaceutical workforce planning, which requires improved collaboration and participation of academic, practice, professional and governmental sectors, would create a regional environment for impactful workforce transformation. A commitment in the WHO Eastern Mediterranean Region would build on the four strategic objectives of WHO’s *Framework for action for health workforce development in the Eastern Mediterranean Region* developed to implement the *Global strategy on human resources for health*.^8^ This commitment would also support the operationalization of the Amman Commitment to take action on pharmaceutical primary health-care reform for this region.^12^ The current workforce transformation programme rolling out in Jordan^4^ is a good example of how the need to identify, address and monitor workforce trends, needs and progression are having ground-level impact in service provision.

## Conclusion

Pharmacists are a vital human resource for health care that is increasingly being harnessed to contribute to the global health agenda of UHC and equitable access to health services, particularly to primary care. Strengthening and advancing the capacity of the pharmacy workforce is therefore an integral strategy for enhancing health system performance and keeping with WHO’s strategy of no health care without a workforce. This strategy underscores the need for a capable and knowledgeable pharmacy workforce possessing the necessary skills relevant for population needs. The Global Advanced Development Framework provides a tool for professional development of the pharmaceutical health workforce and facilitates the availability of flexible, effective and advanced practice pharmacists equipped to meet the needs of UHC across countries. The framework can be adapted by other countries to local needs and for advancing and transforming the national pharmaceutical health workforce.
